# Demographic characteristics and type/frequency of physical activity participation in a large sample of 21,603 Australian people

**DOI:** 10.1186/s12889-018-5608-1

**Published:** 2018-06-05

**Authors:** Rochelle M. Eime, Jack T. Harvey, Melanie J. Charity, Rayoni Nelson

**Affiliations:** 1 School of Health Sciences and Psychology, Federation University, Ballarat, Australia; 2 Institute of Sport, Exercise and Active Living, Victoria University, Melbourne, Australia; 3 Victorian Health Promotion Foundation (VicHealth), Melbourne, Australia

**Keywords:** Sport, Leisure-time physical activity, Demographic correlates

## Abstract

**Background:**

Regular physical activity (PA) is imperative for good health and there are many different ways that people can be active. There are a range of health, PA and sport policies aiming to get more people active more often. Much research has been directed towards understanding the determinants of inactivity and PA. However, it is important to understand the differences not only between inactive and active people, but also between activity contexts (for example participation in sport compared to non-sport activities), in order to align policies and strategies to engage market segments who have different participation preferences and accessibility. The aim of this study was to investigate demographic correlates of the propensity to be physically inactive or active within different contexts, and at different levels of frequency of participation.

**Methods:**

Data from the Australian Exercise, Recreation and Sport Survey was used for this analysis. This included information on the type, frequency and duration of leisure-time PA for Australians aged 15 years and over. Reported PA participation in the two-week period prior to the survey was used to allocate respondents into three categories: no PA, non-sport PA only, and sport. Subsequently, sport participants were further categorised according to frequency of participation. Potential demographic correlates included sex, age, education, employment, marital status, language spoken, having a condition that restricts life, children, and socio-economic status.

**Results:**

The survey included 21,603 people. Bivariate chi-squared analysis showed that there were significant differences between the profiles of leisure-time PA participation across all demographic variables, except the variable languages spoken at home. Ordinal regression analysis showed that the same demographic variables were also correlated with the propensity to engage in more organised and competitive PA contexts, and to participate more frequently.

**Conclusions:**

People who were female, older, married or had a disability were less likely to participate in sport. Therefore when designing PA opportunities to engage those who are inactive, particularly those that are organised by a club or group, we need to ensure that appropriate strategies are developed, and tailored sport products offered, to ensure greater opportunities for increased diversity of participation in sport.

## Background

Being physically active is important for overall health including physical and mental health [1]. Regular physical activity (PA) can improve fitness and contribute to lower risk of chronic diseases such as coronary heart disease, stroke, some cancers, type 2 diabetes, osteoporosis and depression [1]. Despite the wide ranging benefits of being physically active we have a global inactivity epidemic which contributes to an overweight and obesity health crisis [2, 3], amongst other chronic health conditions. World-wide 31% of adults are physically inactive, and 80% of 13–15 year olds are not meeting the recommended levels of PA or 60 min of moderate to vigorous intensity per day [3].

People can be physically active in different PA domains or contexts, including home, work, transport and leisure-time [4]. The context of leisure-time PA has three aspects to it, which have been defined as the type, mode and setting [5]. “Type” refers to the specific activity (e.g. football, athletics, swimming). Different modes of participation include team sports (e.g. football, cricket and netball), individual sports (e.g. tennis, athletics and triathlon), organised but non-competitive PA (e.g. cycling and running groups), and non-organised or informal PA (e.g. going to the gym or a walk) [5]. Settings of participation include organisational settings, such as schools, clubs or leisure centres, and neighbourhood settings such as home, street or park [5].

Within the PA guidelines and it is recognised that there are different health gains through different types of activities such as aerobic or endurance and muscle-strengthening or resistance training, [1]. There is also growing body literature that highlights that there are different types and levels of health gain according to the domain or context of participation [6–9]. For example, participation in sport, can be associated with improved social and mental health above and beyond improvements attributed to individual types of PA, because of the social nature of team and club-based participation [6, 7, 9].

From a policy perspective, there are a range of health and PA policies aimed at getting more people more active and more often in order to promote healthier individuals, communities and nations [10–12]. There are clear and relatively consistent recommendations internationally, including in the UK, USA and Australia, for duration of engagement in moderate to vigorous intensity PA: at least 60 min per day for children and young people, and at least 30 min on at least 5 days per week (i.e. 2.5 h weekly) for adults [1, 13, 14].

With regard to sport in particular, policies in countries including England and Australia are very consistent and clear that the aim is to get more people active and keep them active. “We are seeking a consistent increase in the proportion of people regularly playing sport” – Sport England [15]. The task of the Australian Sports Commission (ASC) is also to get more people playing sport more often, and with a specific focus on young Australians [16]. In the Australian context there are government synergies between sport and health, with sport being located within health portfolios at both the national and Victorian state levels. More specifically, the primary focus of the Victorian Health Promotion Foundation (VicHealth) is “promoting good health and preventing chronic disease” [17] and their Action Agenda for Health Promotion 2013 to 2023 includes the priority of encouraging regular PA. As part of their PA, sport and walking investment plan (2014–2018) there is a whole-of-population approach to getting the inactive and somewhat active people to become more active [10]. This approach is about shifting participation levels along the continuum from inactive to somewhat active to regularly active and very active [10]. Furthermore, their recent PA strategy (2018–2023) uses research evidence to focus on specific priority target groups and with reference to particular action areas or key determinants associated with those specific groups of the population [10]. These approaches consider ways to incorporate PA into everyday life through active living (active travel), active recreation (social, non-competitive PA during leisure time) and organised sport (competitive sport) [10].

Much research has been conducted on the demographic determinants of sedentary behaviour [18, 19] and PA [4, 20] to inform policies and strategies for getting people physically active. There is some research specifically relating to the demographic determinants of sport participation [21–24]. However, the definition of sport is quite variable internationally. For example, in Australia sport is defined as ‘A human activity capable of achieving a result, requiring physical exertion and/or physical skill which, by its nature and organisation, is competitive and is generally accepted as being a sport’. The ASC maintains the final authority for determining whether an activity meets the definition of a sport [25]. In contrast, countries such as England have a broader definition which encompasses physical recreation generally [26]. Sport England’s strategy encompasses both traditional team sports and activities such as walking or going to the gym [26].

It is important that we understand the differences not only between inactive and active people, but also between the active people within different participation contexts (types, modes and settings), and at different levels of frequency, to align policies and strategies to engage specific market segments who have different participation preferences and levels of access to participation opportunities [27, 28]. Accordingly, the aim of this study was to investigate demographic correlates of the propensity to be physically inactive or active within different PA contexts, and at different levels of frequency of participation. The demographic correlates included personal characteristics (sex, age, education, employment, marital status, languages spoken, disability, dependent children) and measures of community socioeconomic status and remoteness.

## Methods

### Participants and procedure

Data from the Australian Exercise, Recreation and Sport Survey (ERASS) were used for this analysis. Details of ERASS methods have been previously described [29, 30]. Briefly, ERASS collected information on the type, frequency and duration of leisure-time PA for Australians aged 15 years and over. Telephone interviewers collected data on PA for two time frames for each respondent, with different characteristics recorded for the ‘previous two weeks’ time frame and the ‘last 12 months’ period. For example, duration of activity was recorded for the ‘previous two weeks’ period, while level of organisation of activity (leisure centre, club, etc.) was recorded for the 12 month data.

In addition to details of PA, ERASS collected demographic data from all respondents surveyed; not just those that were physically active. Questions included age, sex and postcode along with characteristics such as cultural background, education level and employment status.

This multi-wave cross-sectional national survey was conducted in four quarterly tranches each year from 2001 to 2010, and data were weighted by state, region (metropolitan or rest of the state), age group, gender and year to reduce response bias in sample estimates. [31].

### Measures

Self-reported participation in various PA activities in the two-week period prior to the survey was used to allocate respondents into categories for analysis. The first allocation was based on frequency or duration of participation in PA over the two-week time-frame. Categories were: No PA; Non-sport PA only; and Sport. The second allocation incorporated frequency of sport participation as a representation of ‘dose’ of sport-based PA. The ‘Sport’ category above was divided into two further categories based on frequency of participation in any sport. The resulting ordinal ‘dose’ categories were: No PA; Non-sport PA only; Sport 1–3 times per fortnight; and Sport 4+ times per fortnight. Respondents who reported a mixture of sport and non-sport PA were included in the ‘Sport’ categories for both allocations.

Potential demographic correlates of PA participation included sex, age, education, employment, marital status, speaking a language other than English at home, having a condition that restricts life, having children under 18 living at home, and an areal measure of socio-economic status (SES) - quintile of the Socio-economic Indexes for Areas (SEIFA) Index of Relative Socio-economic Advantage and Disadvantage (IRSAD) for postcode of residence [32] (1 = most disadvantaged to 5 = most advantaged).

### Data analysis

First, chi-squared tests of independence were used to investigate bivariate associations between the category of PA undertaken and each demographic characteristic in turn. Second, because the three categories of PA were considered to be naturally ordered with respect to the associated ‘dose’ of PA, ordinal logistic regression was used to investigate the association between the propensity to participate in ‘higher-dose’ vs ‘lower-dose’ ordered categories of PA and each demographic characteristic in turn. Ordinal regression predicts the odds of being in higher-dose versus lower-dose categories, averaged across all possible dichotomies derived from the ordered categories, in this case “no PA vs any PA”, and “no PA or non-sport PA” vs sport PA. This was implemented in a single multivariate model, with the effect of each demographic characteristic on the odds being adjusted for the effects of all other demographic characteristics.

Similar analyses were conducted with PA participation ‘dose’ further subdivided into four ordinal categories: No PA; Non-sport PA only; Sport 1–3 times per fortnight; and Sport 4+ times per fortnight.

## Results

In 2010, 21,603 people were surveyed regarding their participation in leisure-time PA. Approximately 82% of the sample (*n* = 17,769) stated they did some form of PA over the past 12 months, while 18% (*n* = 3834) stated they did none. When those that had done PA in the past 12 months were asked about PA in the past two weeks, 15,049 (85%) indicated they did some form of PA while 2637 (15%) did not, and 83 did not respond.

The age for PA participants in 2010 ranged from 15 to 96 years, with a mean of 44.0 and a standard deviation of 18.5 years.

### Bivariate analyses

The breakdown of those who did some form of leisure-time PA in the past two weeks across various demographic variables is shown in the first part of Table 1. Bivariate chi squared analysis showed that there were significant differences between the profiles of PA participation (no PA, non-sport PA, sport) across all of the demographic variables except languages spoken at home. However, it should be borne in mind that because of the very large sample size and consequent high statistical power, the tests of significance are very sensitive to small differences in the profiles of participation. Because all of the cross-tabulations have more than two categories in one or both dimensions, the details of the differences in the response profiles are complex. In the following paragraph we summarise some key differences, focusing on the likelihood of playing sport.

**Table 1:** Associations between demographic characteristics and type of PA participation in the past two weeks

	Cross-tabulation (Bivariate)									Ordinal logistic regression (Multivariate)		
	No PA		Non-sport PA		Sport PA		Total	*p*-value*		OR	95% CI	*p*-value**
Predictor	*n*	%	*n*	%	*n*	%						
Sex	2720	15.3	7382	41.5	7667	43.1	17,769	< 0.001				< 0.001
Male	1359	15.2	2852	32.0	4699	52.7	8910			ref		
Female	1361	15.4	4530	51.1	2968	33.5	8859			0.53	0.48–0.58	
Age Range	2696	15.4	7248	41.3	7608	43.3	17,552	< 0.001				< 0.001
15–29 years	766	16.9	1149	25.4	2609	57.7	4524			ref		
30–49 years	1097	16.4	2816	42.2	2766	41.4	6678			0.70	0.59–0.83	
50+ years	834	13.1	3283	51.7	2233	35.2	6349			0.62	0.54–0.72	
Education	2720	15.3	7382	41.5	7667	43.1	17,769	< 0.001				0.001
< Year 12, still at school	856	17.1	1911	38.3	2227	44.6	4994			ref		
Highest level of secondary school	577	15.7	1503	40.9	1598	43.5	3678			0.98	0.85–1.12	
Undergraduate diploma, Certificate or Trade qualification	589	15.7	1686	44.9	1484	39.5	3760			0.99	0.87–1.13	
University degree or higher	698	13.1	2282	42.7	2358	44.2	5338			1.22	1.08–1.38	
Employment	2703	15.3	7312	41.5	7614	43.2	17,629	< 0.001				0.008
Full time	1332	16.7	3022	37.9	3628	45.5	7981			ref		
Part time	576	14.4	1711	42.8	1711	42.8	3999			1.18	1.05–1.33	
Other***	796	14.1	2579	45.7	2274	40.3	5649			1.15	1.03–1.29	
Marital status	2705	15.3	7343	41.5	7631	43.2	17,678	< 0.001				< 0.001
Not married	1009	13.9	2723	37.4	3546	48.7	7278			ref		
Married (includes defacto)	1696	16.3	4619	44.4	4085	39.3	10,400			0.79	0.72–0.87	
Language spoken at home	2720	15.3	7382	41.5	7667	43.1	17,769	0.492				0.196
English	2407	15.2	6612	41.8	6802	43.0	15,820			ref		
Other than English	313	16.1	771	39.5	865	44.4	1949			0.90	0.77–1.05	
Has condition that restricts life	2717	15.3	7377	41.6	7660	43.1	17,754	< 0.001				< 0.001
No	2283	14.8	6186	40.1	6956	45.1	15,425			ref		
Yes	434	18.7	1191	51.1	704	30.2	2329			0.64	0.57–0.72	
Number of children aged under 18 at home	2716	15.3	7381	41.6	7664	43.2	17,761	0.002				0.031
None	1777	14.5	5055	41.2	5430	44.3	12,263			ref		
One	348	18.7	835	44.9	677	36.4	1860			0.80	0.67–0.96	
Two	379	16.1	958	40.6	1021	43.3	2358			1.06	0.9–1.24	
Three or more	212	16.5	533	41.6	536	41.8	1281			1.00	0.81–1.22	
SEIFA IRSAD 2011	2717	15.3	7381	41.6	7665	43.2	17,764	0.005				0.574
quintile 1 (Most disadvantaged)	512	17.3	1205	40.8	1233	41.8	2949			ref		
quintile 2	565	16.3	1346	38.9	1552	44.8	3463			1.10	0.94–1.28	
quintile 3	563	15.6	1555	43.0	1495	41.4	3613			0.98	0.85–1.13	
quintile 4	559	15.4	1472	40.4	1610	44.2	3640			1.03	0.89–1.19	
quintile 5 (Most advantaged)	518	12.7	1804	44.0	1775	43.3	4097			1.06	0.92–1.22	

Notes: *OR* odds ratio, *CI* Confidence interval, *ref* Reference category; * Chi-squared analysis; ** Ordinal regression; ***Other = unemployed+not in labour force

Males were much more likely to play sport than females, with 52.7% of males playing sport compared to 33.5% of females. Conversely, 51.1% of females were more likely to report only non-sport PA, compared to 32.0% of males. Those in the younger age range were the most likely to play sport; 57.7% of 15–29 year olds played sport, while only 35.2% of those 50 and over reported playing sport. Those with an undergraduate diploma, certificate or trade qualification were least likely to play sport (39.5%), while in all other education categories, the rate of sport participation was around 44%. Regarding employment, participants who were employed full-time were the most likely to play sport (45.5%), with the proportion diminishing with the level of employment. Those who were not married were more likely to play sport, with 48.7% playing sport compared to 39.3% of those that were married. Those who have a condition that restricts life were less likely to play sport; 45.1% of those with no restrictive condition played sport, while only 30.2% of those with a condition played sport. Language spoken at home was not a significant correlate of the level of PA participation. The number of children aged under 18 at home and SEIFA IRSAD quintile were significant correlates, but in each case there was no clear trend in the profiles of participation across the categories of the predictor.

### Ordinal regression analysis

The second part of Table 1 shows the results of multiple ordinal logistic regression models for predicting the likelihood (represented by the ‘odds’) of a person being in a ‘higher dose’ category of PA engagement vs being in any of the ‘lower dose’ categories, averaged across the PA ‘dose’ categories. The effect of each demographic variable in turn on these odds is represented by a set of ‘odds ratios’, with each odds ratio representing the difference in the odds in the particular demographic category relative to the odds in a chosen ‘reference category’ (the first category listed). The odds ratios are also adjusted for the effects of the incidental changes in all other demographic variables.

After controlling for other demographic variables, sex (*p* < 0.001), age (*p* < 0.001), education level (*p* = 0.001), employment status (*p* = 0.008), marital status (*p* < 0.001), having a condition that restricts life (*p* < 0.001) and having children living at home (*p* = 0.031) had significantly different participation profiles. As expected, being female (OR 0.53), older (30–49 OR 0.70, 50 plus OR 0.62), married (0.79) or having a disability (OR 0.64) made people less likely to participate in higher dose levels of PA and sport than people in the respective reference category. Those having part-time work or not being in the labour force were shown to be more likely to participate in higher dose levels of PA (part time OR 1.18, not in labour force OR 1.15). Results indicate that people with one child were less likely to participate in higher dose levels of PA while having two or more children was no different to having no children in terms of participation in higher dose levels of PA (one child OR 0.80, two children OR 1.06, three or more children OR 1.00, *p* = 0.031).

So for example, after adjustment for other demographic factors, the odds of females being in a higher dose category of PA are significantly less than the odds of males being in a higher dose category of PA (OR = 0.53, 95% CI = 0.48–0.58). Similarly, after adjustment for other demographic factors, the odds of those aged 30–49 years being in a higher dose category of PA are significantly less than the odds of those aged 15–29 years being in a higher dose category of PA (OR = 0.70, 95% CI = 0.59–0.83). Again, after adjustment for other demographic factors, the odds of those with a university degree or higher qualification being in a higher dose category of PA are significantly greater than the odds of those still at school being in a higher dose category of PA (OR = 1.22, 95% CI = 1.08–1.38). However, the odds of those whose highest educational level is completion of secondary school being in a higher dose category of PA are not significantly different than the odds for those still at school (OR = 0.98, 95% CI = 0.85–1.12).

Table 2 shows the results of similar analyses, but with the third category of activity (sport participation) split into two categories on the basis of frequency of sport participation to produce four ordinal categories as the outcome variable. Of the four PA levels (no PA, non-sport, sport 1–3 times per fortnight, 4+ times per fortnight) participants were generally most likely to participate in non-sport PA (approximately 15, 42, 11 and 32% respectively).

**Table 2:** Associations between demographic characteristics and type/frequency of PA participation in the past two weeks

	Cross-tabulation											Ordinal logistic regression		
	No PA		Non-sport PA		Sport 1–3 times per fortnight		Sport 4+ times per fortnight		Total	*p*-value*		OR	95% CI	*p*-value**
Predictor	*n*	%	*n*	%	*n*	%	*n*	%						
Sex	2720	15.3	7382	41.5	2025	11.4	5642	31.8	17,769	< 0.001				< 0.001
Male	1359	15.2	2852	32.0	1300	14.6	3399	38.1	8910			ref		
Female	1361	15.4	4530	51.1	725	8.2	2244	25.3	8859			0.55	0.50–0.60	
Age Range	2696	15.4	7248	41.3	2013	11.5	5595	31.9	17,552	< 0.001				< 0.001
15–29 years	766	16.9	1149	25.4	678	15.0	1932	42.7	4524			ref		
30–49 years	1097	16.4	2816	42.2	835	12.5	1931	28.9	6678			0.71	0.60–0.83	
50+ years	834	13.1	3283	51.7	500	7.9	1732	27.3	6349			0.65	0.57–0.75	
Education	2720	15.3	7382	41.5	2025	11.4	5642	31.8	17,769	< 0.001				< 0.001
< Year 12, still at school	856	17.1	1911	38.3	673	13.5	1554	31.1	4994			ref		
Highest level of secondary school	577	15.7	1503	40.9	432	11.7	1167	31.7	3678			1.02	0.89–1.16	
Undergraduate diploma,Certificate/Trade qualification	589	15.7	1686	44.9	403	10.7	1081	28.8	3760			1.04	0.92–1.18	
University degree or higher	698	13.1	2282	42.7	518	9.7	1841	34.5	5338			1.32	1.17–1.48	
Employment	2703	15.3	7312	41.5	2012	11.4	5602	31.8	17,629	< 0.001				< 0.001
Full time	1332	16.7	3022	37.9	1120	14.0	2508	31.4	7981			ref		
Part time	576	14.4	1711	42.8	352	8.8	1359	34.0	3999			1.27	1.13–1.42	
Other***	796	14.1	2579	45.7	539	9.5	1735	30.7	5649			1.20	1.08–1.34	
Marital status	2705	15.3	7343	41.5	2020	11.4	5610	31.7	17,678	< 0.001				< 0.001
Not married	1009	13.9	2723	37.4	912	12.5	2634	36.2	7278			ref		
Married (includes defacto)	1696	16.3	4619	44.4	1109	10.7	2976	28.6	10,400			0.80	0.73–0.88	
Language spoken at home	2720	15.3	7382	41.5	2025	11.4	5642	31.8	17,769	0.695				0.119
English	2407	15.2	6612	41.8	1792	11.3	5010	31.7	15,820			ref		
Other than English	313	16.1	771	39.5	233	12.0	632	32.4	1949			0.89	0.76–1.03	
Has condition that restricts life	2717	15.3	7377	41.6	2022	11.4	5638	31.8	17,754	< 0.001				< 0.001
No	2283	14.8	6186	40.1	1847	12.0	5108	33.1	15,425			ref		
Yes	434	18.7	1191	51.1	174	7.5	529	22.7	2329			0.65	0.58–0.74	
Number of children aged under 18 at home	2716	15.3	7381	41.6	2025	11.4	5639	31.8	17,761	< 0.001				0.022
None	1777	14.5	5055	41.2	1342	10.9	4088	33.3	12,263			ref		
One	348	18.7	835	44.9	218	11.7	459	24.7	1860			0.79	0.67–0.93	
Two	379	16.1	958	40.6	318	13.5	703	29.8	2358			1.02	0.88–1.19	
Three or more	212	16.5	533	41.6	147	11.5	389	30.4	1281			1.00	0.82–1.21	
SEIFA IRSAD 2011	2717	15.3	7381	41.6	2023	11.4	5642	31.8	17,764	0.001				0.509
quintile 1 (Most disadvantaged)	512	17.3	1205	40.8	379	12.8	854	29.0	2949			ref		
quintile 2	565	16.3	1346	38.9	447	12.9	1105	31.9	3463			1.10	0.95–1.27	
quintile 3	563	15.6	1555	43.0	389	10.8	1106	30.6	3613			1.00	0.87–1.15	
quintile 4	559	15.4	1472	40.4	411	11.3	1198	32.9	3640			1.05	0.92–1.20	
quintile 5 (Most advantaged)	518	12.7	1804	44.0	397	9.7	1378	33.6	4097			1.09	0.95–1.25	

*OR* odds ratio, *CI* Confidence interval, *ref* Reference category, * Chi-squared analysis; ** Ordinal regression; ***Other = unemployed+not in labour force

After controlling for other demographic variables participants were less likely to do PA at a higher dose level if they were female (OR 0.55, *p* < 0.001), older (30–49 OR 0.71, 50+ OR 0.65, *p* < 0.001), married (OR 0.80, *p* < 0.001) or having a restrictive health condition (OR 0.65, *p* < 0.001). Those with a university degree or higher were more likely to participate in PA at a higher dose level (OR 1.32, *p* < 0.001). Those having part-time work or not being in the labour force were shown to be more likely to participate in PA at a higher dose level (part time OR 1.27, not in labour force OR 1.20, *p* < 0.001). Results indicate that people with one child were less likely to participate in higher dose levels of PA while having two or more were no different than having none in terms of participation in higher dose levels of PA (one child OR 0.79, two children OR 1.02, three or more children OR 1.00, *p* = 0.022).

## Discussion

This study provides information on demographic correlates across the PA dosage spectrum from no-leisure-time PA to sport. This is described by VicHealth as the range of ways to incorporate PA in to everyday life to encourage the inactive and somewhat active to become more active, including through active living, active recreation and organised sport [10]. Furthermore, it includes an examination of frequency of participation, which is important from a health perspective.

More than 80% of survey respondents to ERASS had participated in some PA within the past 12 months, and within the past two weeks. However this does not mean that they are active at ‘healthy’ or health-enhancing levels. A recent study using the same dataset explored the health-enhancing levels of PA participation. Overall, 94% of the different types of PA were classified as health enhancing, and 18% of these activities were club-based sport [30]. Furthermore, most (78%) of the Health Enhancing Levels of PA sport participation was played regularly [30]. The Australian rates of PA within the past two weeks is higher than those in the European Union, which used a broad sport definition including both sport and recreation. In this study participation ranged considerably across 11 countries from participating at least once a week of 22% in Portugal to 76% in Finland [21]. England reports participation rates of adult (16+) at 40% at least once a week from 2005 to 2006 [21]. As these authors acknowledged, it is difficult to look at international comparisons when there are major differences in definitions and survey designs [21].

It is well acknowledged that population levels of frequent PA are low and that an improved understanding of the characteristics of people who are inactive and somewhat active can assist development and implementation of strategies for widespread participation in PA and sports [4, 33]. Furthermore sport policies must strive to make sports available to everyone and counter inequality and difference, and therefore sport programs need to be designed more specifically for target groups [33].

This study shows that a number of demographic variables are correlated with a proxy indicator of “dose” of PA. Specifically, being female, older, married, having a restrictive condition, being employed full-time, having a lower level of education, having a child under 18 at home, and living in a lower SES area are all associated with a lower likelihood of participating in higher dose contexts. For education, those with a degree are more likely to be active and active at the higher dose levels. Many studies have investigated the dose-response of participation in different domains of PA and all-cause mortality [34, 35]. However many of these do not consider the actual domain of PA, and they do not investigate the demographical correlates, with the exception of sometimes age and gender [35]. A systematic review and meta-analysis of studies of the general population did investigate participation in different domains of PA and reported that there was stronger associations between PA and all-cause mortality for women than for men, and for sport and leisure-time PA than for occupational and transport related PA [34]. The authors conclude that these differences may be due to differences in the intensity of participation [34].

Sex and age were the main factors relating to PA in a recent Spanish study [36]. Males engaged in more vigorous PA and light PA overall, whereas females performed more moderate PA [36]. Similarly a study of demographic determinants of participation in Sport (and recreation) in Spain and England reported that gender, age, occupation and education level were significant factors in both countries [21]. However the sports participation rate was higher in England 48% compared to Spain 37% and there were demographic differences. The gender difference in participation in England was much lower than that in Spain (11% against 16% respectively) [21], while the age effect was more pronounced in England: education effects were also more important in Spain [21].

An Australian study utilising the same ERASS dataset as the present study found that participation in sport and PA was related to SES, in that the rates of ‘any recreational PA in the past year’ and ‘regular PA’ both increased as SES increased (being areas of greater advantage), however that participation in PA was only SES-prohibitive for only a few types of PA [29]. As SES decreased (being areas of greater disadvantage), participation in many teams sports actually increased [29]. Other sports studies have investigated the relationship between SES and access to facilities, with the hypothesis that socially disadvantaged communities may experience further contextual disadvantage with less access to sports facilities [37]. A German study also investigated the associations between facility provision and disadvantage [37]. This study included free and fee-based facilities and reported that for children and adolescents a lower SES area was actually related to a higher availability of PA facilities [37].

The correlates investigated in this study are generally non-modifiable, so we need to look beyond the correlate itself. Another study of the individual correlates of PA also report that age, sex, health status, self-efficacy and motivation are associated with being active [4]. It may be that females in general and those older, married and with lower education have lower self-efficacy and motivation which may hinder their participation. A systematic review study of the determinants of PA maintenance reported that the difference between individuals who did and did not maintain participation in PA over time reported that maintainers had stronger self-efficacy and intention compared to relapsers [20]. That is, the beliefs about capabilities and motivation and goals were the strongest predictors of participation [20]. More specifically related to sport, the Sport Commitment Model is an evolving theory that explains participation in PA the sport context [38, 39]. Satisfaction and enjoyment and personal investments are consistent predictors of commitment to persistent participation in PA in the form of sport and exercise [38].

An important limitation of this study is the fact that the ERASS was limited to persons aged 15+ years of age, whereas in the case of many sports, children and adolescents younger than 15 years of age constitute a large proportion of participants. However, the Australian Sports Commission’s newly developed national population tracking survey, AusPlay, includes provision for each adult respondent living with a child or children aged 0–14 to answer questions about one randomly selected child. Consequently, future studies of PA participation will be able to cover all ages across the lifespan.

## Conclusion

This study has shown that a number of individually significant demographic correlates of participation in PA are also correlated with the propensity to engage in more organised and competitive PA contexts, and that also relate to participating more frequently. People who were female, older, married or had a disability were less likely to participate in sport. These demographic correlates, captured in the ERASS survey and investigated in this study, are largely non-modifiable. We also need to consider how to improve those that are modifiable, such as self-efficacy, competency and motivation to be physically active, which can be addressed by providing a participation environment which is supportive, social, fun and that allows for different ability and skill levels. In terms of commitment to participate, there are differences between the requirements of club-based sport and unorganised PA that need to be considered. There are also often different motivational factors relating to participation in club-based sport compared to individually-based unorganised PA. Therefore when designing PA opportunities to engage those who are inactive, particularly those that are organised by a club or group, we need to develop the sporting opportunities at clubs from the traditional competitive only model of play. Instead, we need to ensure that appropriate strategies are developed, and tailored sport products offered, to ensure greater opportunities for increased diversity of participation in sport.

## Abbreviations

ASC: Australian Sports Commission
ERASS: Exercise Recreation and Sport Survey
IRSAD: Index of Relative Socio-Economic Advantage and Disadvantage
PA: physical activity
SEIFA: Socio-Economic Indexes for Areas
SES: Socio-Economic Status

## References

[1] US Department of Health and Human Services. Physical activity guidelines for Americans. Washington, DC: Office of Disease Prevention and. Health Promotion. 2008;

[2] Smith GD (2016). A fatter, healthier but more unequal world. Lancet.

[3] Hallal PC, Andersen LB, Bull FC, Guthold R, Haskell W, Ekelund U (2012). Lancet physical activity series working G. Global physical activity levels: surveillance progress, pitfalls, and prospects. Lancet.

[4] Bauman AE, Reis RS, Sallis JF, Wells JC, Loos RJF, Martin BW (2012). Correlates of physical activity: why are some people physically active and others not?. Lancet.

[5] Eime RM, Harvey JT, Sawyer NA, Craike MJ, Symons CM, Polman RC, Payne WR (2013). Understanding the contexts of adolescent female participation in sport and physical activity. Res Q Exerc Sport.

[6] Eime R, Young J, Harvey J, Charity M, Payne W (2013). A systematic review of the psychological and social benefits of participation in sport for adults: informing development of a conceptual model of health through sport. Int J Behav Nutr Phy..

[7] Eime R, Young J, Harvey J, Charity M, Payne W (2013). A systematic review of the psychological and social benefits of participation in sport for children and adolescents: informing development of a conceptual model of health through sport. Int J Behav Nutr Phy.

[8] Basterfield L, Reilly J, Pearce M, Partkinson K, Adamson A, Reilly J, Vella S (2015). Longitudinal associations between sports participation, body composition and physical activity from childhood to adolescence. J Sci Med Sport.

[9] Vella S, Cliff D, Magee C, Okley A (2015). Associations between sports participation and psychological difficulties during childhood: a two-year follow up. J Sci Med Sport.

[10] VicHealth. Physical activity, sport and walking (2014). VicHealth's Investment Plan (2014 to 2018).

[11] Australian Sports Commission: Play. Sport. Australia. The Australian Sports Commission's participation game plan. Canberra, Australia: Australian Sports Commission. 2015;26.

[12] Sport and Recreation Victoria: Active Victoria - A strategic framework for sport and recreation in Victoria 2017–2021. 2017. http://sport.vic.gov.au/publications-and-resources/strategies/active-victoria-strategic-framework-sport-and-recreation. Accessed 27 Apr 2018.

[13] Physical activity guidelines for adults. 2011. https://www.gov.uk/government/uploads/system/uploads/attachment_data/file/213740/dh_128145.pdf. Accessed 2 Mar 2018.

[14] Department of Health. Australia’s physical activity and sedentary Gudelines. Canberra: Australian Government; 2014.

[15] Department of Culture, Media and Sport. Creating a sporting habit for life A new youth sport Strategy London: Sport England; 2012: 20.

[16] Australian Sports Commission: Corporate plan 2016–20. Australian Sports Commission; 2016: 48.

[17] We aim to create a Victoria where everyone can enjoy better health 2016. https://www.vichealth.vic.gov.au/about/what-we-do. Accessed 2 Mar 2018.

[18] Chastin SFM, Buck C, Freiberger E, Murphy M, Brug J, Cardon G, O’Donoghue G, Pigeot I, Oppert J-M. Systematic literature review of determinants of sedentary behaviour in older adults: a DEDIPAC study. Int J Behav Nutr Phy. 2015;12(1) 10.1186/s12966-015-0292-3 PMC4595239 26437960

[19] O’Donoghue G, Perchoux C, Mensah K, Lakerveld J, van der Ploeg H, Bernaards C, Chastin SFM, Simon C, O’Gorman D, Nazare J-A (2016). A systematic review of correlates of sedentary behaviour in adults aged 18–65 years: a socio-ecological approach. BMC Public Healt.

[20] Amireault S, Godin G, Vézina-Im L-A (2013). Determinants of physical activity maintenance: a systematic review and meta-analyses. Health Psychol Review.

[21] Kokolakakis T, Lera-López F, Panagouleas T (2012). Analysis of the determinants of sports participation in Spain and England. Appl Econ.

[22] Federico B, Falese L, Marandola D, Capelli G (2012). Socioeconomic differences in sport and physical activity among Italian adults. J Sport Sci.

[23] Downward P, Rasciute S (2015). Exploring the covariates of sport participation for health: an analysis of males and females in England. J Sport Sci..

[24] Eime RM, Casey MM, Harvey JT, Sawyer NA, Symons CM, Payne WR (2015). Socioecological factors potentially associated with participation in physical activity and sport: a longitudinal study of adolescent girls. J Sci Med Sport.

[25] ASC Recognition. Undated. https://www.ausport.gov.au/supporting/nso/asc_recognition. Accessed 2 Mar 2018.

[26] Sport England our Strategy 2017. https://www.sportengland.org/active-nation/our-strategy/. Accessed 2 Mar 2018.

[27] Australian Sports Commission. Market segmentation for sport participation- adults. March. Canberra: Australian Sports Commission; 2013.

[28] Australian Sports Commission (2013). Market segmentation for sport participation: children. Canberra: Australian sports commission.

[29] Eime RM, Charity MJ, Harvey JT, Payne WR (2015). Participation in sport and physical activity: associations with socio-economic status and geographical remoteness. BMC Public Health.

[30] Eime R, Harvey J, Charity M, Casey M, van Uffelen J, Payne W (2015). The contribution of sport participation to overall health enhancing physical activity levels in Australia: a population-based study. BMC Public Health.

[31] Standing Committee on Recreation and Sport. Participation in exercise, recreation and sport. In. Canberra: Australian Sports Commission; 2010:186.

[32] Australian Bureau of Statistics Socio-economic indexes for areas (SEIFA). Canberra: Australian bureau of Statistics; 2013.

[33] Puig N (2016). The sports participation: from research to sports policy. Physical culture and sport studies and research.

[34] Samitz G, Egger M, Zwahlen M (2011). Domains of physical activity and all-cause mortality: systematic review and dose-response meta-analysis of cohort studies. Int J Epidemiol.

[35] Hupin D, Roche F, Gremeaux V, Chatard J-C, Oriol M, Gaspoz J-M, Barthélémy J-C, Edouard P (2015). Even a low-dose of moderate-to-vigorous physical activity reduces mortality by 22% in adults aged≥ 60 years: a systematic review and meta-analysis. Brit J Sport Med.

[36] Mielgo-Ayuso J, Aparicio-Ugarriza R, Castillo A, Ruiz E, Avila J, Aranceta-Batrina J, Gil A, Ortega R, Serra-Majem L, Varela-Moreiras G (2016). Physical activity patterns of the Spanish population are mostly determined by sex and age: findings in the ANIBES study. PLoS One.

[37] Schneider S, D'Agostino A, Weyers S, Diehl K, Gruber J (2015). Neighborhood deprivation and physical activity facilities—no support for the deprivation amplification hypothesis. JPAH.

[38] Williams L: Commitment to sport and exercise. Re-examining the literature for a practical and parsimonious model. J Prev Med Public Health 2013;46 Suppl 35–42. 10.3961/jpmph.2013.46.S.S35 PMC3567317 23412904

[39] Scanlan T, Carpenter P, Simons J, Schmidt G, Keeler B (1993). The sport commitment model: measurement development for the youth-sport domain. J Sport Exercise Psy.

